# *Ehrlichia* spp. and *Anaplasma* spp. in Xenarthra mammals from Brazil, with evidence of novel ‘*Candidatus* Anaplasma spp.’

**DOI:** 10.1038/s41598-020-69263-w

**Published:** 2020-07-28

**Authors:** Ana Cláudia Calchi, Juliana Gaboardi Vultão, Mario Henrique Alves, Débora Regina Yogui, Arnaud Leonard Jean Desbiez, Mariele De Santi, Matheus de Souza Santana, Thiago Merighi Vieira da Silva, Karin Werther, Marta Maria Geraldes Teixeira, Rosangela Zacarias Machado, Marcos Rogério André

**Affiliations:** 1 Laboratório de Imunoparasitologia, Departamento de Patologia Veterinária, Faculdade de Ciências Agrárias e Veterinárias Júlio de Mesquita Filho (UNESP), Campus de Jaboticabal, Via de Acesso Prof. Paulo Donato Castellane, s/n, Zona Rural, Jaboticabal, São Paulo CEP: 14884-900 Brazil; 2 Instituto de Ciências Biomédicas, Universidade de São Paulo (USP), São Paulo, SP Brasil; 3 ICAS-Instituto de Conservação de Animais Silvestres-Projeto Bandeiras e Rodovias, Campo Grande, Mato Grosso Do Sul (MS) Brasil

**Keywords:** Bacteria, Parasitology

## Abstract

Anaplasmataceae agents are obligatory intracellular Gram-negative α-proteobacteria that are transmitted mostly by arthropod vectors. Although mammals of the Superorder Xenarthra (sloths, anteaters, and armadillos) have been implicated as reservoirs for several zoonotic agents, only few studies have sought to detect Anaplasmataceae agents in this group of mammals. This study aimed to investigate the occurrence and genetic diversity of *Anaplasma* spp. and *Ehrlichia* spp. in blood and spleen samples of free-living Xenarthra from four different states in Brazil (São Paulo, Mato Grosso do Sul, Rondônia, and Pará). Nested and conventional PCR screening assays were performed to detect the *rrs* and *dsb* genes of *Anaplasma* spp. and *Ehrlichia* spp., respectively. The assays were positive in 27.57% (91/330) of the *Anaplasma* spp. and 24.54% (81/330) of the *Ehrlichia* spp. Of the 91 positive *Anaplasma* spp. samples, 56.04% were positive in a conventional PCR assay targeting the 23S–5S intergenic region. Phylogenetic and distance analyses based on the *rrs* gene allocated *Anaplasma* sequences from sloths captured in Rondônia and Pará states in a single clade, which was closely related to the *A. marginale*, *A. ovis,* and *A. capra* clades. The sequences detected in southern anteaters from São Paulo were allocated in a clade closely related to sequences of *Anaplasma* spp. detected in *Nasua nasua*, *Leopardus pardalis*, and *Cerdocyon thous* in Brazil. These sequences were positioned close to *A. odocoilei* sequences. Genotype analysis corroborated previous findings and demonstrated the circulation of two distinct *Anaplasma* genotypes in animals from north and southeast Brazil. The first genotype was new. The second was previously detected in *N. nasua* in Mato Grosso do Sul state. The intergenic region analyses also demonstrated two distinct genotypes of *Anaplasma*. The sequences detected in Xenarthra from Pará and Rondônia states were closely related to those in *A. marginale*, *A. ovis,* and *A. capra*. *Anaplasma* spp. sequences detected in Xenarthra from São Paulo and were allocated close to those in *A. phagocytophilum*. The analyses based on the *dsb* gene grouped the *Ehrlichia* spp. sequences with sequences of *E. canis* (São Paulo, Mato Grosso do Sul, and Pará) and *E. minasensis* (Rondônia and Pará). The data indicate the occurrence of *E. canis* and *E. minasensis* and two possible new *Candidatus* species of *Anaplasma* spp. in free-living mammals of the Superorder Xenarthra in Brazil.

## Introduction

The Anaplasmataceae family (order Rickettsiales) is formed by the genera *Anaplasma*, *Ehrlichia*, *Neorickettsia,* and *Wolbachia*^1^. They are comprised of obligatory intracellular Gram-negative bacteria, that transmit mostly through arthropod vectors, mainly ticks. These agents can infect different mammalian cells depending on the infecting species. *A. marginale*, *A. centrale,* and *A. ovis* infect erythrocytes; *A. platys* infects platelets and neutrophils; *A. odocoilei* infects platelets; *A. phagocytophilum* and *E. ewingii* infect granulocytes; *A. bovis*, *E. chafeensis*, *E. canis*, *E. muris,* and *Neorickettsia* spp. infect monocytes and macrophages; *E. ruminantium* infects endothelial cells, neutrophils, and macrophages; and *Wolbachia* spp. is an invertebrate endosymbiont^1,2^. They form microcolonies (morulas) in intracytoplasmic vacuoles^3^. Domestic and wild animals are considered reservoirs, and favor the spread of these agents that can cause diseases in animals and humans^1,3,4^.

In Brazil, several genotypes of *A. phagocytophilum*,* A. marginale*,* A. bovis*,* A. platys*,* E. canis*,* E. chafeensis*, and potential new species, that are not yet named, have been detected in different species of wild animals, including deer^5–11^, wild canids^12–14^, wild felids^12,15,16^, coatis^14^, rodents^14,17–19^, caititus and peccaries^20^, opossums^21,22^, and birds^23,24^.

The Xenarthra superorder is composed of two orders: Cingulata and Pilosa. Cingulata is characterized by the presence of a bone carapace. A representative animal is the armadillo. Pilosa features a dense coat and the absence or underdevelopment of teeth. Representative animals are sloths and anteaters^25,26^. These animals are distributed from the central southern region of North America to southern of South America^26^. They are considered reservoirs of several zoonotic agents^27^.

Studies on Anaplasmataceae agents in Xenarthra are scarce. *Anaplasma marginale* was detected in a giant anteater (*Myrmecophaga tridactyla*) in Argentina by blood smears and molecular confirmation by PCR assays based on the *msp-5* and *msp1-α* genes^28^. Genotypes of *Ehrlichia* spp., based on the *dsb* gene, and *Anaplasma* spp., based on the 16S rRNA gene, were detected in a three-toed sloth (*Bradypus tridactylus*) in the state of Pará in northern Brazil^30^.

The present study aimed to investigate the occurrence and genetic diversity of *Anaplasma* spp. and *Ehrlichia* spp. in Xenarthra mammals sampled in four different Brazilian states.

## Material and methods

### Ethical statement

Animal procedures and management protocols were approved by the Institute Chico Mendes for Conservation of Biodiversity (SISBIO N º 53798-5) and by the Ethics Committee on Animal Use of the Biomedical Sciences Institute—University of São Paulo (protocol number 98) and the School of Agricultural and Veterinarian Sciences (FCAV/UNESP; protocol number 11.794). All methods and experimental protocols were performed in accordance with the relevant FCAV/UNESP Ethics Committee guidelines and regulations.

### Sampling and sampled species

**Figure 1:**
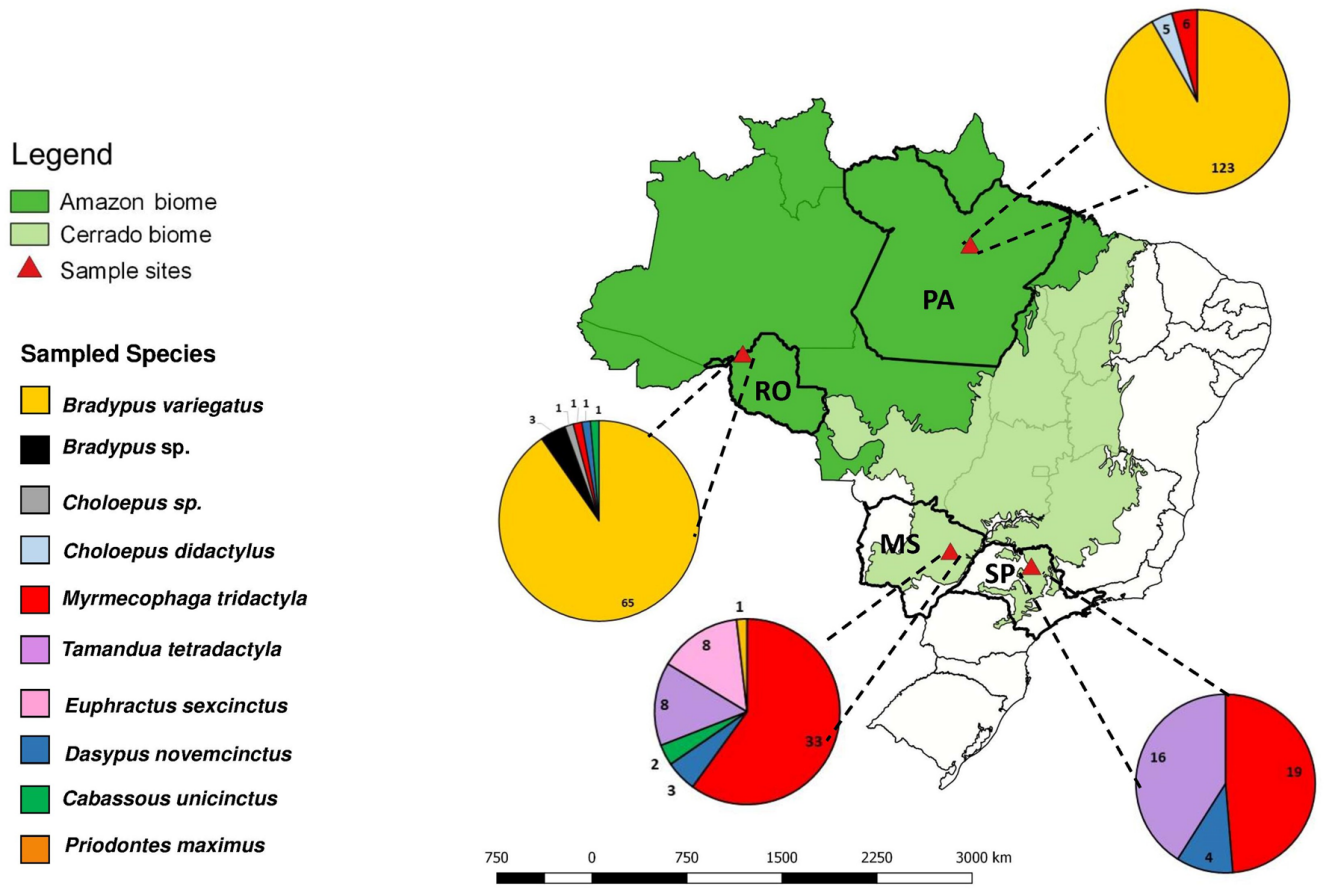
Number and origin of the mammals from the Superorder Xenarthra sampled in Brazil. *SP* São Paulo, *MS* Mato Grosso do Sul, *PA* Pará, *RO* Rondonia. The map was created using QGIS v.3.10.5 software (https://www.qgis.org/en/site/forusers/download.html.).

The biological samples of Xenarthra mammals originated from four Brazilian states: Mato Grosso do Sul (MS), São Paulo (SP), Pará (PA), and Rondônia (RO). The total of 330 animals included 188 brown-throated sloths (*Bradypus variegatus*), three *Bradypus* spp., five two-toed sloths (*Choloepus didactylus*), 31 *Choloepus* spp., 31 southern anteaters (*Tamandua tetradactyla*), 52 Giant Anteaters (*Myrmecophaga tridactyla*), three southern naked-tailed armadillos (*Cabassous unicinctus*), eight nine-banded armadillos (*Dasypus novemcinctus*), eight six-banded armadillo (*Euphractus sexcinctus*), and one giant armadillo (*Priodontes maximus*) (Fig. 1).

Spleen samples were collected by the Bandeiras e Rodovias Project team and by the Wildlife Pathology Service of FCAV/UNESP during necropsies of road-killed animals in the Cerrado biome in Mato Grosso do Sul (20 °26′ 48.3″ S 52° 54′ 11.6″ W) and São Paulo (21° 17′ 33.2″ S 48° 19′ 53.4″ W). In the state of Mato Grosso do Sul, 55 spleen samples (33 giant anteaters, eight southern anteaters, eight six-banded armadillos, three southern nine-banded armadillos, one giant armadillo, and two southern naked-tailed armadillos) were collected during 2017 and 2018. In São Paulo state, 39 spleen samples (19 giant anteaters, 16 southern anteaters, and four southern nine-banded armadillos) were obtained from 2011 to 2018. In addition, 236 blood samples were collected by cephalic vein venipuncture from sloths, armadillo and anteaters during the filling process of a hydroelectric power plant in Amazonia biome in the states of Rondônia (3° 07′ 20.2″ S 51° 46′ 31.5″ W) (n = 102, comprising 65 brown-throated sloths, 31 *Choloepus* spp., three Bradypus spp., one giant anteater, one nine-banded armadillo, and one southern naked-tailed armadillo) and Pará (9° 16′ 21.1″ S 64° 37′ 59.2″ W) (n = 134, comprising 123 brown-throated sloths, five two-toed sloths, and six giant anteaters).

Spleen fragments and blood samples were stored in KASVI^®^ RNAse and DNAse-free microtubes at − 70 °C until DNA extraction.

### Molecular analysis

**Figure 2:**
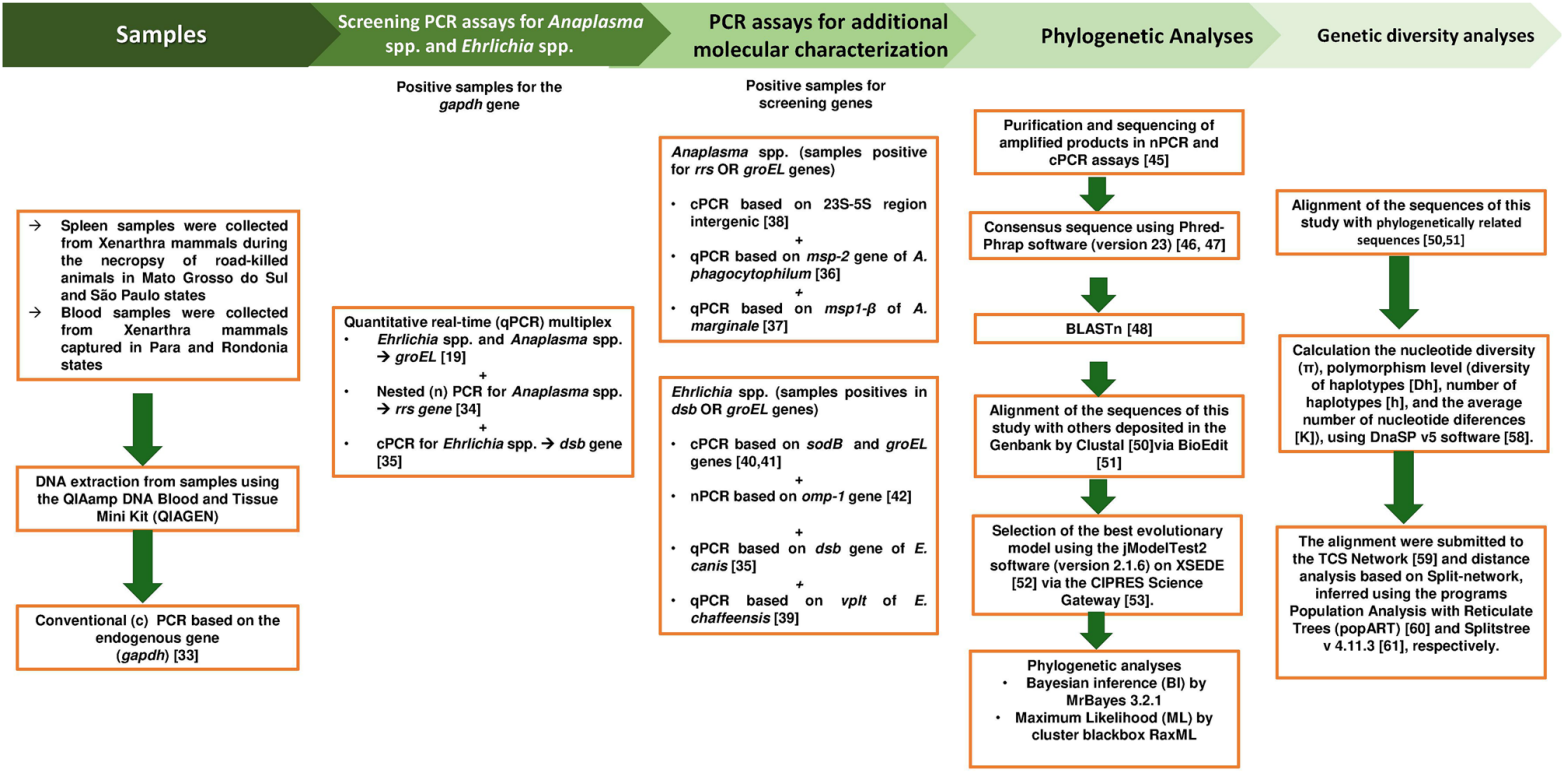
Workflow with the steps performed in the methodology of this study.

DNA was extracted from 10 mg of each spleen tissue and 200 μL of each blood sample using the DNeasy Blood & Tissue Kit (Qiagen, Valencia, CA, USA), according to the manufacturer’ s instructions. The presence of amplifiable DNA was verified by a conventional PCR (cPCR) assay targeting the mammalian endogenous glyceraldehyde-3-phosphate dehydrogenase (*gapdh*) gene^29^. The positive samples were subjected to specific PCR assays for *Anaplasma* spp. and *Ehrlichia* spp. (Fig. 2).

#### *Anaplasmaspp.detection*

Screening for *Anaplasma* spp. based on the *groEL* and *rrs* genes was performed using multiplex qPCR (quantitative real-time PCR)^18^ and nested PCR (nPCR)^30^ protocols, respectively (Tables 1, 2). The positive samples, for at least one of the two protocols, were tested by qPCR assays based on the *msp-2*^31^ and *msp1-β*^32^ genes for *A. phagocytophilum* and *A. marginale*, respectively (Table 1). cPCR assays for *Anaplasma* spp. based on the 23S–5S intergenic region were also performed^33^ (Table 2).

**Table 1:** Description of primers, hydrolysis probes and thermal sequences used in qPCR assays for *Ehrlichia* spp. and *Anaplasma* spp.

Agents (target-genes)	Primers	Hydrolysis probe (TaqMan)	Thermal sequences	References
*Ehrlichia* spp. (*groEL* gene)^a^	5′-GCGAGCATAATTACTCAGAG-3’ 5′-CAGTATGGAGCATGTAGTAG-3’	TET-5′-CATTGGCTCTTGCTATTGCTAAT- 3′[BHQ2a-Q]3′	− 95 °C for 3 min; 40 cycles: 95 °C for 10 min and 52.8 °C for 30 s	^18^
*Anaplasma* spp. (*groEL* gene)^a^	5′-TTATCGTTACATTGAGAAGC-3′ 5′GATATAAAGTTATTAAAAGTATAAAGC-3′	Cy-5-5′-CCACCTTATCATTACACTGAGACG-3′[BHQ2a-Q]3′	− 95 °C for 3 min; 40 cycles: 95 °C for 10 min and 52.8 °C for 30 s	^18^
*E. canis *(*dsb* gene)	5′-TTGCAAAATGATGTCTGAAGATATGAAACA-3′ 5′-GCTGCTCCACCAATAAATGTATCYCCTA-3′	5′ FAM AGCTAGTGCTGCTTGGGCAACTTTGAGTGAA-[ BHQ-1–3′]	95 °C for 5 min, 40 cycles: 95 °C for 15 s and 60 °C for 1 min	^34^
*E. chaffeensis *(*vlpt* gene)	5′-CTAATTCTGATTTACACGAGTCTTC-3′ 5′-GCATCATCTTCGAATTGAACTTC-3′	5′[TAMRA] (TTGAGTGTCC[BHQ2a-Q]3′	95 °C for 3 min, 40 cycles: 95 °C for 10 min e 55 °C for 30 s	^35^
*A.phagocytophilum* (*msp-2* gene)	5′-AGTTTGACTGGAACACACCTGATC-3 5′-CTCGTAACCAATCTCAAGCTCAAC-3′	5′[FAM] (939p-TTAAGGACAACATGCTTGTAGCTATGGAAG-GCA-[TAMRA])	50 °C for 2 min, 95 °C for 10 min, 40 cycles: 95 °C for 15 s and 60 °C for 1 min	^31^
*A. marginale *(*msp1-β* gene)	5′-TTGGCAAGGCAGCAGCTT-3′ 5′-TTCCGCGAGCATGTTGCAT-3′	5′[FAM] (TCGGTCTAACATCTCCAGGCTTTCAT-3′-BHQ1	95 °C for 10 min, 40 cycles: 95 °C for 15 s and 60 °C for 1 min	^32^

^a^qPCR multiplex.

**Table 2:** Description of primers, amplicon sizes and thermal sequences used in conventional and nested PCR assays for *Ehrlichia* spp. and *Anaplasma* spp. Protocols marked with * represent nested PCR assays.

Agents	Primers sequences	Size (bp)	Thermal sequences	References
*Anaplasma* spp. (*rrs* gene)* External primers gE3a gE10R Internal primers gE2 gE9f	5′-CACATGCAAGTCGAACGGATTATTC-3′ 5′-TTCCGTTAAGAAGGATCTAATCTCC′-3′ 5′-GGCAGTATTAAAAGCAGCTCCAGG-3′ 5′-AACGGATTATTCTTTATAGCTTGCT-3′	932 546	94 °C for 5 min 40 cycles: 94 °C for 30 s, 55 °C for 30 s and 72 °C for 1 min 72 °C for 5 min	^30^
*Ehrlichia* spp. (*dsb* gene) dsb-330 (F) dsb-728 (R)	5′- GATGATGTCTGAAGATATGAAACAAAT-3′ 5′-CTGCTCGTCTATTTTACTTCTTAAAGT-3′	409	95 °C for 2 min; 50 cycles: 95 °C for 15 s, 58 °C for 30 s and 72 °C por 30 segundos 72 °C for 5 min	^34^
*Anaplasma *spp. (ITS—23S–5S) * ITS2F* ITS2R	5′-AGGATCTGACTCTAGTAACGAG-3′ 5′-CTCCCATGTCTTAAGACAAAG-3′	300	94 °C for 2 min, 35 cycles; 94 °C for 30 s, 58 °C for 30 s, 72 °C for 1 min 72 °C for 5 min	^33^
*Ehrlichia *spp. (*groEL* gene) groEL124-F1 groEL808-R1	5′-ATTAAGCCAGAAGAACCATTAGC-3′ 5′-TACTGCAATATCACCAAGCATATC-3′	680	95 °C for 5 min, 40 cycles: 95 °C for 30 s, 54 °C for 30 s and 72 °C for 30 s 72 °C for 5 min	^36^
*Ehrlichia *spp*. *(*sodB* gene) * sodbEhr1600-F* * sodbEhrl600-R*	5′-ATGTTTACTTTACCTGAACTTCCATATC-3′ 5′-ATCTTTGAGCTGCAAAATCCCAATT-3′	600	94 °C for 3 min; 55 cycles: 94 °C for 10 s; 58 °C for 10 s; 72 °C por 15 s 72 °C for 30 s 72 °C for 5 min	^37^
*Ehrlichia* spp. (*omp-1* gene)* External primers * conP28-F1* * conP28-R1* Internal primers * conP28-F2* * conP29-R2*	5′AT(C/T)AGT(G/C)AAA(A/G)TA(T/C)(A/G)T(G/A)CCAA-3′ 5′TTA(G/A)AA(A/G)G(C/T)AAA(C/T)CT(T/G)CCTCC-3′ 5′CAATGG(A/G)(T/A)GG(T/C)CC(A/C)AGA(AG)TAG-3′ 5′TTCC(T/C)TG(A/G)TA(A/G)G(A/C)AA(T/G)TTTAGG-3′	300	94 °C for 3 min, 35 cycles: 94 °C for 1 min, 50 °C for 1 min and 72 °C for 2 min 72 °C for 5 min	^38^

#### *Ehrlichiaspp.detection*

Screening for *Ehrlichia* spp. based on *groEL* and *dsb* genes was performed using multiplex qPCR (quantitative real-time PCR)^18^ and cPCR^34^ protocols, respectively (Tables 1, 2). The positive samples, for at least one of the two protocols, were tested by qPCR assays based on *dsb*^34^ and *vlpt*^35^ genes for *E. canis* and *E. chaffeensis*, respectively (Table 1). Additionally, cPCR assays for *Ehrlichia* spp. were based on the *groEL*^36^ and *sodB* genes^37^. In addition, nPCR assays for *Ehrlichia* spp. based on the *omp-*1 gene^38^, were performed (Table 2).

#### *Reactionconditions*

All qPCR assays were performed with a final volume of 10 μL containing 1 μL of DNA sample (concentration mean: 162.5 and 50.55 ng/µL for spleen and blood samples, respectively), 0.2 μM of each primer and hydrolysis probe, 5 μL GoTaq Probe qPCR Master Mix (Promega Corporation, Madison WI, USA), and sterilized ultrapure water (Nuclease-Free Water; Promega Corporation) q.s. 9 μL. PCR amplifications were performed in low-profile multiplate unskirted PCR plates (Bio-Rad, Hercules, CA USA) using a CFX96 Thermal Cycler (Bio-Rad). Quantification of the number of copies of target DNA/μL was performed using IDT psmart plasmids (Integrated DNA Technologies, Coralville, IA, USA) containing the target sequences. Serial dilutions were performed to construct standard curves with different plasmid DNA concentrations (2.0 × 10^7^ to 2.0 × 10^0^ copies/μL). The number of plasmid copies/µL of the amount (g/µL) of DNA/plasmid (bp) was determined by multiplying by 6.022 × 10^23^. Each qPCR assay was performed in duplicate for each DNA sample. All duplicates showing cycle quantification (Cq) values differing by > 0.5 were re-tested. Amplification efficiency (E) was calculated from the slope of the standard curve in each run (E = 10^–1/slope^). The reactions followed the standards established by the Minimum Information for Publication of Quantitative real-time PCR experiments^39^.

All the cPCR assays were performed using 5 μL of the DNA samples (concentration mean: 162.5 and 50.55 ng/µL for spleen and blood samples, respectively) in a mixture containing 1.25 U Platinum Taq DNA Polymerase (Invitrogen, Carlsbad, California, USA), PCR buffer (PCR buffer 10×—100 nM Tris–HCl, pH 9.0, 500 mM KCl), 0.2 mM deoxynucleotides (dATP, dTTP, dCTP, and dGTP) (Invitrogen, Carlsbad, California, United States), 1.5 mM of magnesium chloride (Invitrogen, Carlsbad, CA, United States), 0.5 μM of each primer (Invitrogen), and sterile ultrapure water (Invitrogen) q.s.25 μL. In nPCR assays, 1 μL of the amplified product from the first PCR reaction was used as the target DNA in the second reaction. DNA samples from *A. phagocytophilum,* kindly provided by Prof. John Stephen Dumler (Uniformed Services University of the Health Sciences, Bethesda, MD, USA), and *E. canis*, obtained from DH82 cells infected with the Jaboticabal strain of *E. canis*^40^, were used as positive controls. Sterile ultrapure water (Nuclease-Free Water, Promega Corporation) was used as a negative control. The products were separated by 1% agarose gel electrophoresis at an electric current of 100 V/150 mA for 50 min. The gel was stained with 1% ethidium bromide (Life Technologies, Carlsbad, CA, USA) and examined under ultraviolet light illumination using the ChemiDoc MP Imaging System (Bio-Rad) and photographed using Image Lab Software version 4.1.

The cPCR and nPCR amplified products were purified using the ExoSAP-IT PCR Product Cleanup Reagent (Applied Biosystems, Foster City, CA, USA) and sequenced using the BigDye Terminator v3.1 Cycle Sequencing kit (Thermo Fisher Scientific, Waltham, MA, USA) and the ABI PRISM 310 DNA Analyzer (Applied Biosystems)^41^.

### Phylogenetic analyses

The sequences obtained were submitted to a quality-screening test using Phred-Phrap software (version 23)^42,43^ to evaluate the quality of the electropherograms and to obtain the consensus sequences from the alignment of the sense and antisense sequences. The BLASTn program^44^ was used to compare the obtained nucleotide sequences with previously deposited sequences in the GenBank database^45^.

The consensus sequences obtained in this study and those retrieved from GenBank were aligned using ClustalW software version 7^46^ using Bioedit v. 7.0.5.3^47^. The best evolutionary model was chosen using the jModelTest2 software (version 2.1.6) on XSEDE^48^ via the CIPRES Science Gateway^49^. The phylogenetic analyses were based on Bayesian inference (BI) and maximum likelihood (ML) methods. The BI analyses were performed using MrBayes 3.1.2^50^ via the CIPRES Science Gateway. Markov chain Monte Carlo simulations were run for 10^6^ generations with a sampling frequency of every 100 generations and a 25% burn-in. ML analyses were performed using the Blackbox RaxML cluster^51^ using 1,000 bootstrapping replicates^52^. The phylogenetic trees were edited using TreeGraph 2.0.56-381 beta software^53^.

### Genetic diversity and genealogies

The genetic diversity analyses for the *rrs* gene and 23S–5S intergenic region of *Anaplasma* spp. and for the *dsb* gene of *Ehrlichia* spp. were performed with the sequences obtained in this study aligned to phylogenetically closer sequences of *A. phagocytophilum*, *A. marginale*, *A. ovis*, *A. odocoilei*, *Anaplasma* spp., *E. canis*, *E. minasensis*, and *Ehrlichia* spp. retrieved from GenBank. Clustal/W software^46^ via Bioedit v. 7.0.5.3^47^ was used for the alignment. The sequences used were at least 420 bp, 310 bp, and 310 bp for the *rrs* gene, 23S–5S intergenic region, and *dsb* gene, respectively. Sequences that were smaller in size were excluded from the phylogenetic analysis. These alignments were used to calculate the nucleotide diversity (π), polymorphism level (diversity of haplotypes [Dh], number of haplotypes [h], and the average number of nucleotide differences [K]), using DnaSP v5 software^54^. The sequences were submitted to the TCS Network^55^ and distance analysis based on the split-network was inferred using the programs Population Analysis with Reticulate Trees (popART)^56^ and Splitstree v 4.14.6 ^57^, respectively.

## Results

### Occurrence of Anaplasmataceae agents in Xenarthra mammals

The mean concentration of the extracted DNA was 162.5 ± 37.8 and 50.55 ± 12.1 ng/µL for spleen and blood samples, respectively. All 330 DNA samples were positive in the cPCR assay based on the endogenous *gapdh* gene*.* A total of 147 (44.54%) animals were positive for at least one agent.

Financial restraints limited the selection of samples for sequencing to only a few among the large number of positive samples. The selection was based on two steps. First, we selected samples that presented high intensity amplicons in agarose gel electrophoresis. These samples were then separated according to animal species and region of origin. A random selection was performed to obtain at least one representative of each positive species and of each location.

### *Anaplasma* spp.

Of the 330 samples analyzed, 91 (27.57%) were positive for *Anaplasma* spp. based on the *rrs* gene (Table 3). No sample was positive in the qPCR for *Anaplasma* spp. based on the *groEL* gene. Of the 91 samples positive in the screening assays for *Anaplasma* spp., 51 (56.04%) samples were positive for the 23S–5S intergenic region of *Anaplasma* spp., and 7/91 (7.7%) samples were positive in the qPCR assay for *A. phagocytophilum* based on the *msp-2* gene. The latter positive samples were not quantified because of the low amount of DNA of the agent in the tested samples (Monte Carlo effect)^42^ (Table 3). All samples were negative in the qPCR assays for *A. marginale* (*msp1-β* gene) (Supplementary Table 1).

**Table 3:** Molecular detection of *Anaplasma* spp. and *Ehrlichia* spp. in biological samples (blood or spleen) of Xenarthra mammals.

Xenarthra species (nº sampled)	PCR assays for *Anaplasma* spp.—nº positive (%)/nº positive by state			PCR assays for *Ehrlichia* spp.—nºpositive (%)/nº positive by state
	nPCR screening (*rrs* gene)	cPCR (23S–5S intergenic region)	qPCR (*msp-2* gene)	cPCR screening (*dsb* gene)
*Bradypus variegatus* (188)	65 (34.57%)/34 PA, 31 RO	39 (20.74%)/18 PA, 19 RO	2 (3.07%)/2 PA	56 (29.78%)/42PA, 14RO
*Bradypus* sp. (3)	2 (66.66%)/2 RO	2 (66.6%)/2 RO	0	2 (66.66%)/2 RO
*Choloepus didactylus* (5)	2 (40%)/2 PA	1 (20%)/1 PA	0	1 (20%)/1PA
*Choloepus* sp (31)	13 (41.93%)/13 RO	6 (19.35%)/6 RO	0	5 (16.12%)/5 RO
*Tamandua tetradactyla* (31)	5 (16.12%)/1 PA, 4 SP	3 (9.677%)/3 SP	5 (16.12%)/1PA, 4 SP	10 (32.25%)/1 PA, 4 MS, 5 SP
*Myrmecophaga tridactyla* (52)	0	NT	NT	7 (13.46%)/3 SP, 4 MS
*Cabassous unicinctus* (3)	2 (66.66%)/2 MS	0	0	0
*Dasypus novemcinctus* (8)	1 (12.5%)/1 MS	0	0	0
*Euphractus sexcinctus* (8)	1 (12.5%)/1 MS	0	0	0
*Priodontes maximus* (1)	0	NT	NT	0
Total = 330	91	51	7	81

*PA* Para, *RO* Rondônia, *SP* São Paulo, *MS* Mato Grosso do Sul, *NT* samples not tested due to negative results in the screening PCR assays.

#### *rrsgene*

Of the 91 samples, 25 (27.47%) positive samples for *Anaplasma* spp., based on the *rrs* gene, were sequenced. The sequences obtained were deposited in GenBank under accession numbers MT199810 to MT199833.

BLASTn analysis showed that nine *rrs Anaplasma* sequences obtained from animals sampled in Rondônia and Pará states showed identity ranging from 97.39 to 98.48% with sequences of *A. phagocytophilum* detected in *Haemaphysalis longicornis* (GU064895) and in Chinese water deer (*Hydropotes inermis*; KR611598) sampled in Korea. The 16 remaining sequences of animals from the same states showed identities ranging from 97.71 to 99.38% with *Anaplasma* spp. genotypes detected in *Rattus rattus* from Brazil (KY391803) and in a goat from Saudi Arabia (LC467273). Two sequences detected in Xenarthra sampled from São Paulo state showed identities of 99.04 and 99.50% with *Anaplasma* spp. detected in a Brazilian Brown Brocket Deer (*Mazama gouazoubira*; KF020580) and in a coati (*N. nasua*; KY499186) from Brazil, respectively. One sequence from São Paulo showed 98.69% identity with *A. phagocytophilum* detected in a dromedary from Tunisia (KC455363) (Table 4).

**Table 4:** Percentage of identity assessed by BLASTn of *Anaplasma* and *Ehrlichia* sequences detected in Xenarthra mammals.

Species—ID/localization	Target gene	Query coverage (%)	Identity (%)	GenBank accession numbers
*B. variegatus*—BM6/PA	*rrs*	99	99.35	*Anaplasma* sp.—rodent from Brazil (KY391803)
*B. variegatus*—BM27/PA		100	99.34	
*B. variegatus*—BM31/PA		100	99.35	
*B. variegatus*—BM37/PA		98	99.35	
*C. didactylus*—BM85/PA		100	99.11	
*B. variegatus*—BM117/PA		99	99.33	
*B. variegatus*—BM175/PA		100	99.10	
*B. variegatus*—BM353/PA		98	99.36	
*B. variegatus*—BM415/PA		97	99.36	
*B. variegatus*—PV709/RO		100	98.58	
*Bradypus* sp.—PV1080/RO		100	99.34	
*B. variegatus*—PV1190/RO		99	99.38	
*Choloepus* sp.—PV1225/RO		95	99.36	
*B. variegatus*—BM32/PA	*rrs*	100	98.37	*A. phagocytophilum*—*Haemaphysalis longicornis* from South Korea (GU064895)
*B. variegatus*—BM111/PA		99	98.48	
*B. variegatus*—BM129/PA		99	98.48	
*Choloepus* sp.—PV49/RO		100	98.37	
*B. variegatus*—PV428/RO		100	98.37	
*B. variegatus*—PV442/RO		100	98.29	
*Choloepus* sp.—PV1102/RO		100	98.44	
*B. variegatus*—BM80/PA	*rrs*	99	97.71	*Anaplasma* sp.—goat from Saudi Arabia (LC467273)
*B. variegatus*—BM128/PA	*rrs*	100	97.39	*A. phagocytophilum*—water-deer from Korea (KR611598)
*T. tetradactyla—*60/SP	*rrs*	100	98.69	*A. phagocytophilum* -dromedary from Tunisia (KC455363)
*T. tetradactyla—*90/SP	*rrs*	100	99.04	*Anaplasma* sp. of Brazilian Brown Brocket (KF02058)
*T. tetradactyla—*94/SP	*rrs*	98	99.50	*Anaplasma* sp.—coati (*Nasua nasua*) from Brazil (KY499186)
*B. variegatus—*BM12/PA	Intergenic region (23S–5S)	99	90.20	*A. marginale*—cattle from Brazil (CP023731)
*B. variegatus*—BM31/PA		99	90.97	
*B. variegatus—*BM167/PA		100	90.87	
*B. variegatus—*BM181/PA		100	90.85	
*B. variegatus*—PV14/PA		100	90.89	
*B. variegatus—*PV16/PA		100	90.89	
*B. variegatus—*PV399/RO		100	90.91	
*B. variegatus—*PV737/RO		100	90.89	
*B. variegatus—*PV415/RO		100	90	
*Bradypus* sp.—PV1080/RO		100	90.89	
*Choloepus* sp.—PV1177/RO		100	90.89	
*Choloepus* sp.—PV1206/RO		100	90.89	
*T. tetradactyla*—58/SP	Intergenic region (23S–5S)	100	90.74	*A. phagocytophilum*—sheep from Norway (CP01376)
*T. tetradactyla*—66/SP		99	90.58	
*T. tetradactyla*—NEC17/MS	*dsb*	100	100	*E. canis*—dog from Colombia (MK783026)
*T. tetradactyla*—NEC19/MS		100	100	
*M. tridactyla*—NEC34/MS		100	100	
*M. tridactyla—*NEC63/MS		100	100	
*M. tridactyla*—75/SP		100	100	
*M. tridactyla*—83/SP		100	100	
*D. novemcinctus*—89/SP		100	100	
*B. variegatus*—BM24/PA		100	100	
*B. variegatus—*BM51/PA		100	100	
*B. variegatus*—BM61/PA		100	100	
*T. tetradactyla*—BM177/PA	*dsb*	99	100	*E. minasensis*—cattle from Australia (MH500007)
*B. variegatus—*BM16/PA		100	99.38	
*B. variegatus—*BM180/PA		100	99.70	
*B. variegatus—*PV35/RO		96	99.71	
*B. variegatus—*PV41/RO		87	99.43	
*B. variegatus*—PV14/RO	*dsb*	99	100	*E. minasensis*—*Rhipicephalus microplus* from Brazil (JX629808)
*B. variegatus—*PV726/RO		99	99.47	
*B. variegatus—*PV737/RO		93	98.96	
*Choloepus* sp.—PV1165/RO		99	99.47	

**Figure 3:**
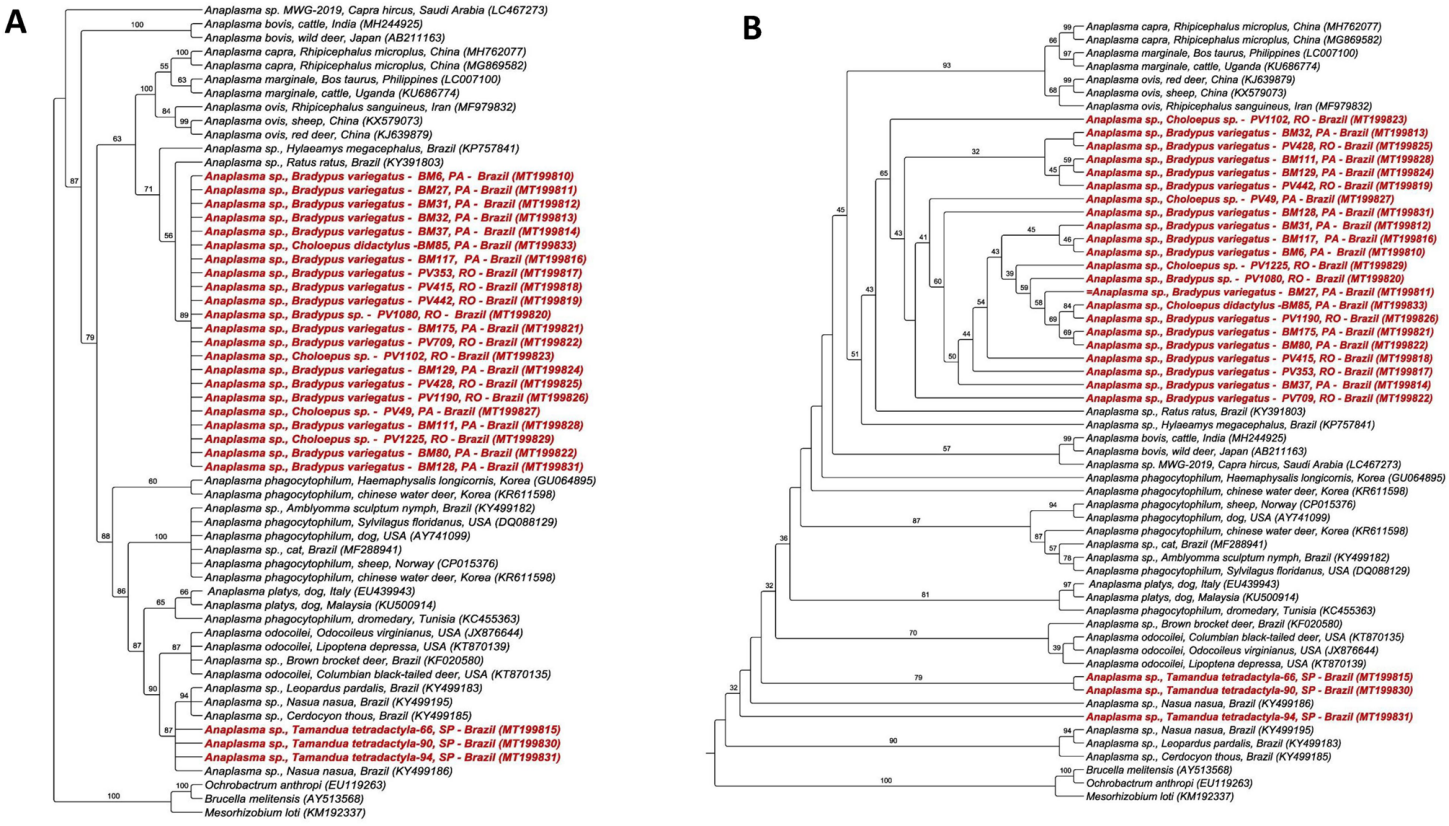
Phylogenetic analysis of *Anaplasma rrs* sequences based on the topology generated by Bayesian model (**A**) and Maximum Likelihood (**B**), with TVM + I + G as evolutionary model. *Ochrobactrum anthropi*, *Brucella melitensis* and *Mesorhzobium Ioti* were used as an external group.

The phylogenetic analysis inferred by the BI method (Fig. 3a) positioned all the sequences obtained in Rondônia and Pará states in a single clade that was phylogenetically closer to *Anaplasma* spp. genotypes detected in rodents in Brazil (KP757841 and KY391803), with 89% branch support. Despite forming a single clade, the obtained sequences showed greater proximity to the clade of *Anaplasma* spp. found in ruminants (*A. capra*, *A. ovis,* and *A. marginale*). On the other hand, the sequences obtained from anteaters of São Paulo state were allocated to a clade closer to the sequences of *Anaplasma* spp. detected in ocelots (*Leopardus pardalis*), coatis (*Nasua nasua*), and crab-eating foxes (*Cerdocyon thous*) from the Pantanal natural region in southern Brazil, with 87% branch support. This clade was sister to the clade of *A. odocoilei*, with 90% branch support. Results of the ML analysis (Fig. 3b) concurred (or agreed) partially with the BI analysis. While the sequences obtained in Rondônia and Pará states formed a single clade close to the clade of *Anaplasma* spp. found in ruminants, the *Anaplasma* sequences obtained from Xenarthra of São Paulo were subdivided into different clades, allocated close to the clade of *A. odocoilei.*

Additionally, four (BM31, BM32, BM128, and BM129) of the seven positive samples for *A. phagocytophilum,* based on the *msp2* gene, were sequenced for the *rrs* gene. Interestingly, three sequences had a high identity with *A. phagocytophilum* by the BLASTn analysis. However, in the phylogenetic analysis, they were positioned together with *rrs* sequences of other *Anaplasma* spp. obtained in Rondônia and Pará states. BLASTn directly presented a greater identity of one sequence (BM31) with *Anaplasma* sp. of *Rattus rattus*.

**Figure 4:**
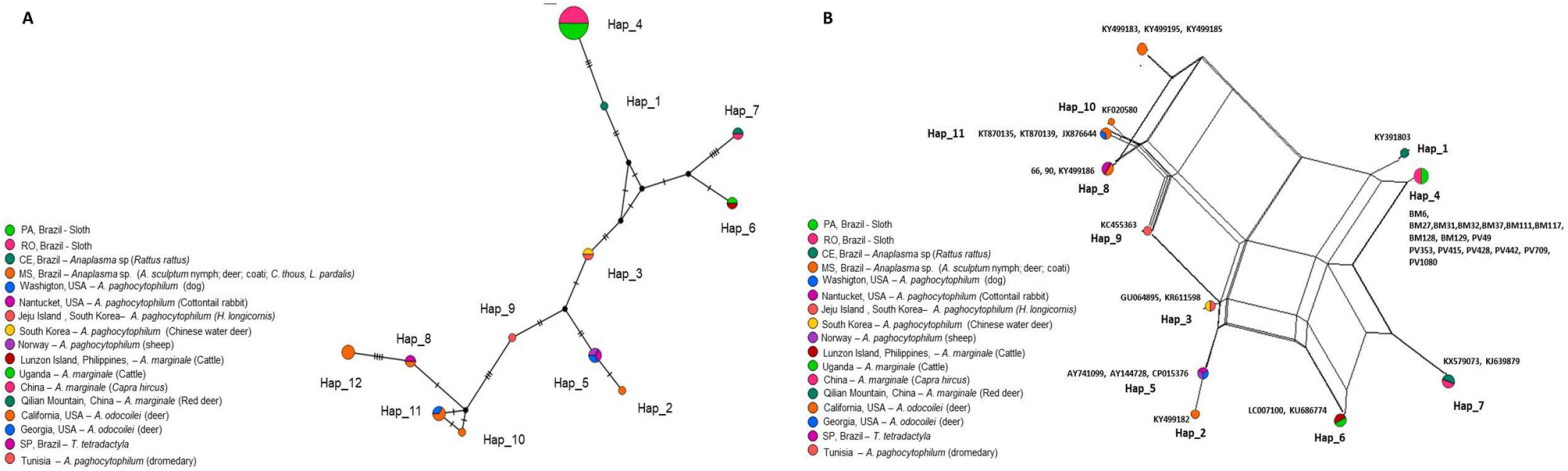
(**A**) TCS network of *rrs* sequences from *A. marginale*, *A. phagocytophilum*, *A. ovis* and those obtained in the present study performed with PopART v.1.7 software (https://popart.otago.ac.nz/index.shtml) (**B**) Split-network performed with Splitstree v4.14.6 software using the parameters “Neighbor-Net and” Uncorrected p-distance (https://en.freedownloadmanager.org/Windows-PC/SplitsTree4-FREE.html.).

Genotype analysis based on 38 *rrs Anaplasma* sequences, including 18 sequences obtained in this study and sequences of *A. marginale, A. ovis, A. phagocytophilum, A. odocoilei,* and *Anaplasma* spp., indicated the presence of 12 genotypes, with π = 0.01719, hd = 0.808, and K = 7.16643 (Table 5, Fig. 4a). Eight genotypes comprised more than one sequence. Genotype #3 comprised two sequences of *A. phagocytophilum* detected in South Korea. Genotype #4 comprised 16 sequences detected in sloths from Rondônia and Pará (this study). Genotype #5 comprised three sequences of *A. phagocytophilum* detected in the United States and Norway. Genotype #6 grouped two sequences of *A. marginale* detected in cattle from the Philippines and Uganda. Genotype #7 grouped two sequences of *A. ovis* detected in sheep and deer in China. Genotype #8 grouped the two sequences detected in anteaters from São Paulo state (this study) and one sequence detected in a coati (*Nasua nasua*) from MS. Genotype #11 comprised three sequences of *A. odocoilei* sequences detected in cervids and a fly (*Lipoptena depressa*) in the United States. Finally, genotype #12 grouped sequences of *Anaplasma* sp. detected in ocelot (*Leopardus pardalis*), crab-eating fox (*Cerdocyon thous*), and coati from Brazil.

**Table 5:** Genetic diversity and polymorphisms of *Anaplasma rrs* and 23S–5S intergenic region and *Ehrlichia dsb* sequences.

Gene/agent	bp	N	VS	GC%	h	dh (mean ± SD)	π (mean ± SD)	K
*rrs/Anaplasma* spp.	417	38	21	0.519	12	0.808 ± 0.059	0.01719 ± 0.00118	7.16643
Intergenic region (23S–5S)/*Anaplasma* spp.	302	23	66	0.472	8	0.719 ± 0.094	0.08454 ± 0.01005	24.09486
*dsb/Ehrlichia* spp.	260	29	19	0.329	4	0.569 ± 0.057	0.0357 ± 0.00221	9.28079

*N* number of sequences analyzed, *VS* number of variable sites, *GC%* C + G content, *h* number of genotypes, *dh* diversity of genotypes, *SD* standard deviation, *π* nucleotide diversity (per site), *K* nucleotide difference number.

Regarding the genotype network (Fig. 4a), it could be inferred that genotype #4, which comprises the *rrs* sequences of *Anaplasma* detected in sloths from Rondônia and Pará states, was derived from genotype #1, which comprises a sequence found in *Rattus* in Brazil (KY391803), upon a mutational event. Both genotypes originated from a median vector (genotype not contemplated in the presented tree). On the other hand, genotype #8, which covers the two sequences obtained in this study in anteaters from São Paulo state and a coati sequence obtained in the state of MS, originated from a median vector upon a mutational event. Moreover, the sequences of *A. odocoilei* also originated from the same median vector. Additionally, genotype #8 gave rise to genotype #12, composed of sequences obtained from different wild animals sampled in the state of MS.

Split-network analysis based on the *rrs* gene (Fig. 4b) corroborated the genotype network, since the Rondônia and Pará sequences were all positioned together and closer to the *Anaplasma* spp. sequence previously detected in *R. rattus*. The phylogenetic analysis revealed that they were positioned closer to the *A. ovis* sequences. The sequences detected in São Paulo were closely positioned to the sequences of *A. odocoilei*.

#### *23S–5Sregionintergenic*

Fourteen (27.45%) of 51 positive samples for *Anaplasma* spp. based on the 23S–5S intergenic region were sequenced. The sequences obtained were deposited in GenBank under access numbers MT267341 to MT267354.

BLASTn analysis showed that 12 sequences obtained in Rondônia and Pará states showed identity ranging from 90 to 90.91% with *A. marginale* detected in a bovine animal from Brazil (CP023731), and two sequences from São Paulo showed 90.58 and 90.74% identity with *A. phagocytophilum* detected in a sheep from Norway (CP015376) (Table 4).

**Figure 5:**
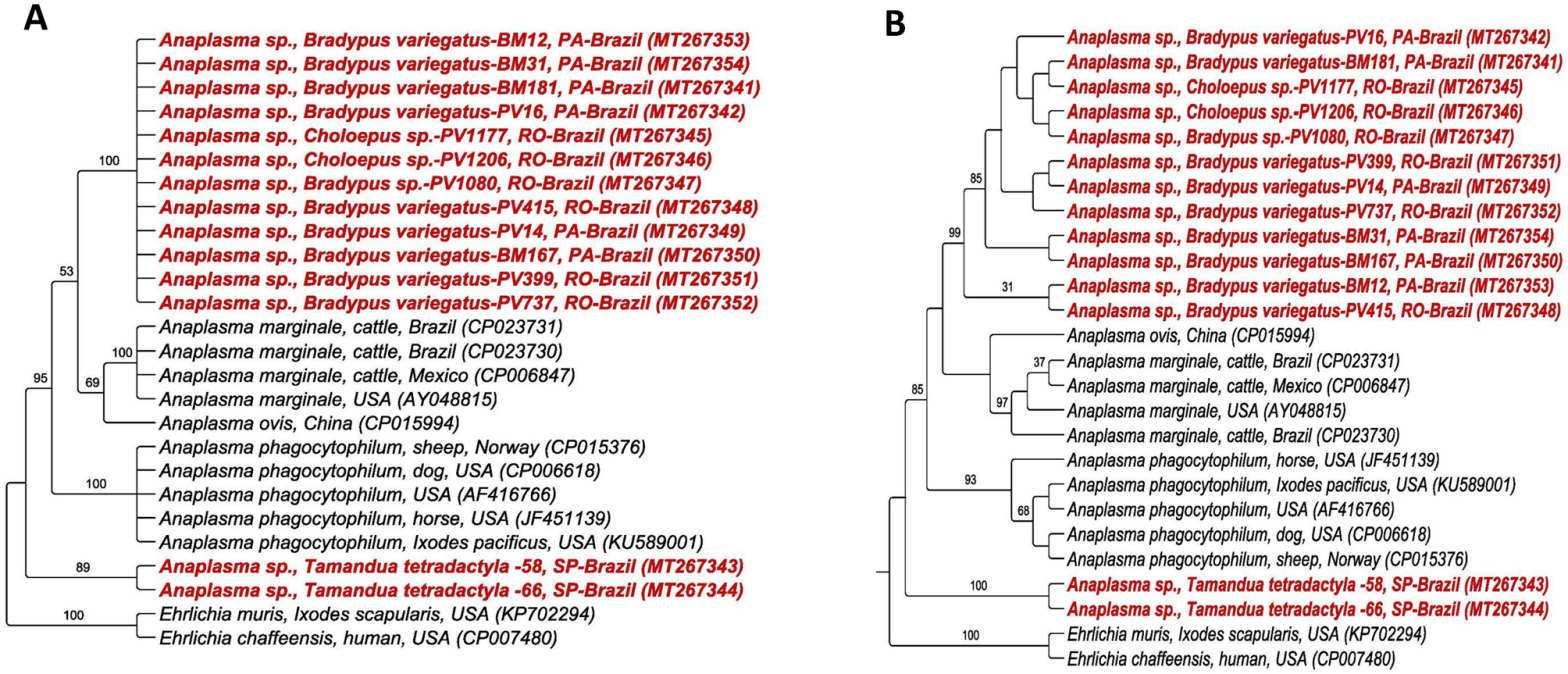
Phylogenetic analysis of *Anaplasma* 23S–5S sequences based on the topology generated by Bayesian (**A**) and Maximum Likelihood (**B**) models, with HKY + G as evolutionary model. *Ehrlichia muris* and *Ehrlichia chaffeensis* were used as an external group.

Phylogenetic analyses based on the 23S–5S intergenic region of *Anaplasma* spp. positioned the sequences detected in Xenarthra in two distinct clades, composed only of sequences found in this study. The first clade was composed of the sequences detected in sloths from the Rondônia and Pará states, which was in close proximity to the clade of *A. marginale* and *A. ovis*. The second clade was composed of sequences obtained from anteaters from SP, which were in close proximity to the clade of *A. phagocytophilum*. Both the BI (Fig. 5a) and ML analyses (Fig. 5b) presented the same topology, although ML presented a better definition of the clades. The index of clade support was 100 and 99% for Rondônia and Pará clade 1 and 89 and 100% for SP clade 2, in the BI and ML analyses, respectively. Of the seven (7.7%) positive samples in the qPCR for the *msp2* gene of *A. phagocytophilum*, only one (14.28%) was positive in the PCR based on the 23S–5S region. However, it was closer to *A. marginale* in both the BLASTn and phylogenetic analyses.

**Figure 6:**
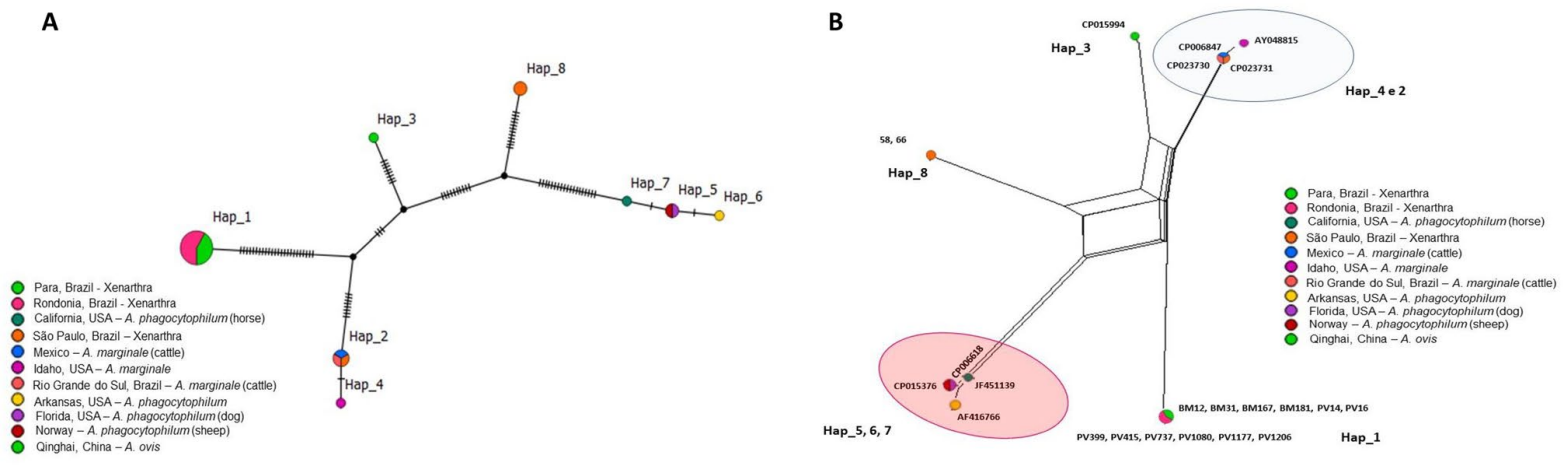
(**A**) TCS network of 23S–5S sequences from *A. marginale*, *A. phagocytophilum*, *A. ovis* and those obtained in the present study performed with PopART v.1.7 software (https://popart.otago.ac.nz/index.shtml) (**B**) Split-network performed with Splitstree v4.14.6 software using the parameters “Neighbor-Net and” Uncorrected p-distance (https://en.freedownloadmanager.org/Windows-PC/SplitsTree4-FREE.html.).

Genotype analysis based on 23 *Anaplasma* 23S–5S sequences, which included 14 sequences obtained from the Xenarthra sampled in this study as well as *A. marginale*, *A. ovis,* and *A. phagocytophilum* sequences, obtained eight different genotypes, with π = 0.08454, hd = 0.719, and K = 24.09486 (Table 5, Fig. 6a). Four genotypes comprised more than one sequence. Genotype #1 comprised all 12 *Anaplasma* 23S–5S sequences from sloths sampled in Rondônia and Pará states. Genotype #8 comprised two sequences detected in giant anteaters from São Paulo state. Genotype #2 comprised three sequences of *A. marginale* previously detected in Brazil and Mexico. Genotype #5 comprised two sequences of *A. phagocytophilum* detected in the United States and Norway. Based on the network of genotypes (Fig. 6a), genotype #1 originated from a median vector and several mutational events. The same median vector also originated from genotypes #2 and #4, corresponding to sequences of *A. marginale*. Genotype #8 also originated from a median vector, which in turn originated from genotype #7, and gave rise to genotypes #5 and 6, comprised of *A. phagocytophilum* sequences.

Corroborating the genotype network findings, the split-network analysis, based on the 23S–5S intergenic region (Fig. 6b), showed a clear separation between the sequences detected in Rondônia and Pará states, and those detected in São Paulo state. Additionally, the Xenarthra sequences from Rondônia and Pará states were closer to the *A. marginale* and *A. ovis* sequences. The Xenarthra sequences from São Paulo were allocated close to *A. phagocytophilum*, corroborating the findings obtained in the other analyses.

### *Ehrlichia* spp.

Of the 330 samples screened for *Ehrlichia* spp., 81 (24.54%) were positive in cPCR assays based on the *dsb* gene (Table 3). No sample was positive in qPCR for *Ehrlichia* spp. based on the *groEL* gene. No samples were positive in the cPCR (*groEL* and *sodB* genes), in the nested PCR (*omp-1* gene) assays for *Ehrlichia* spp., and in the qPCR assays for *E. canis* (*dsb* gene) and *E. chaffeensis* (*vlpt* gene) (Supplementary Table 2).

Nineteen (23.45%) of 81 samples positive for *Ehrlichia* spp. PCR based on the *dsb* gene were sequenced. The sequences obtained were deposited in GenBank under access numbers MT212405 to MT212423.

BLASTn analysis showed that 10 sequences were identical to *E. canis* detected in a dog from Colombia (MK783026). The remaining nine sequences showed identities ranging from 98.96 to 100% with *E. minasensis* detected in *Rhipicephalus microplus* from Brazil (JX629808) and in bovine from Australia (MH500007) (Table 4).

**Figure 7:**
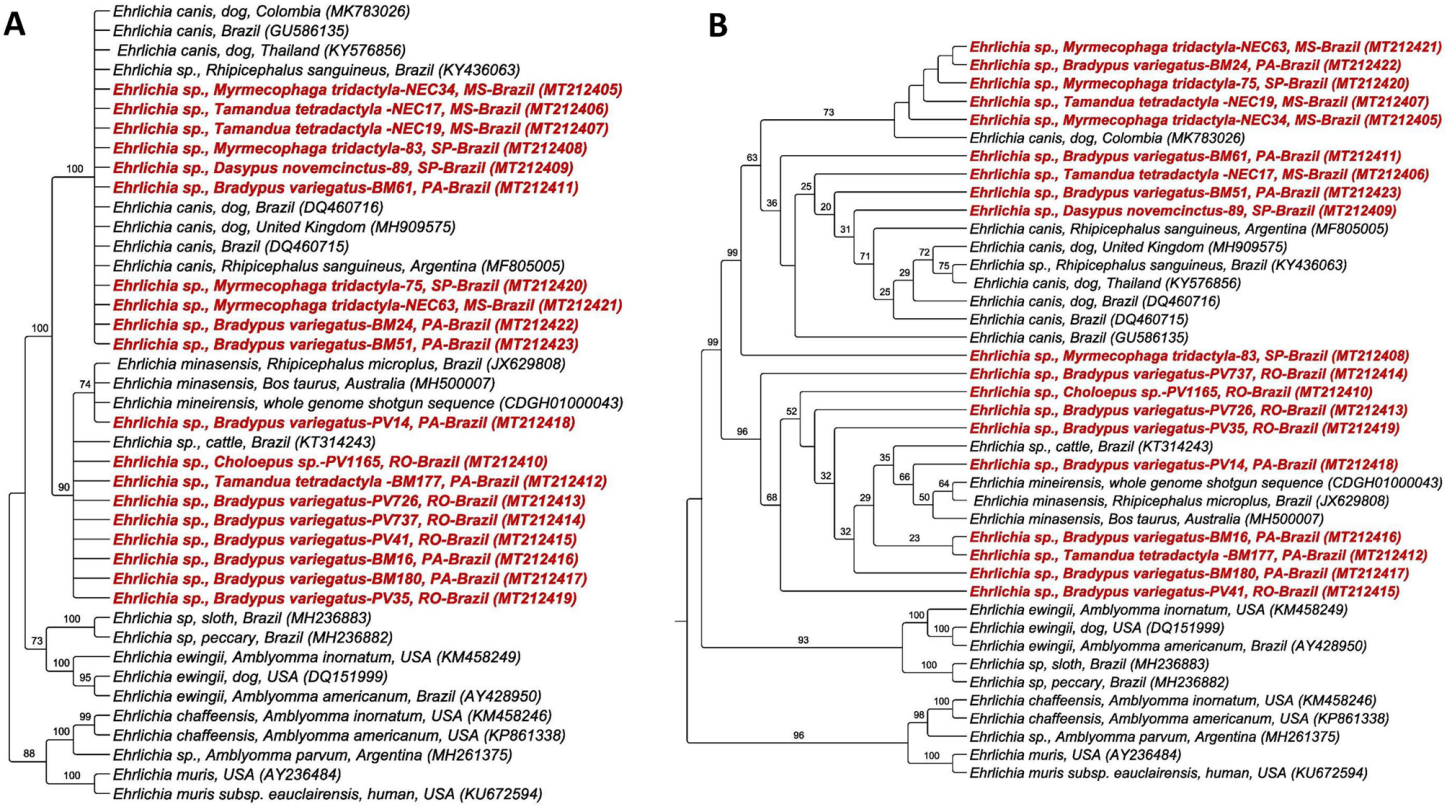
Phylogenetic analysis of *Ehrlichia dsb* sequences based on the topology generated by Bayesian model (**A**) and Maximum Likelihood (**B**), with TrN + I + G as evolutionary model. *Ehrlichia muris* and *Ehrlichia chaffeensis* were used as an external group.

Phylogenetic analyses based on the *dsb* gene of *Ehrlichia* spp. positioned the sequences obtained in this study in the *E. canis* and *E. minasensis* clades. The sequences obtained from São Paulo and Mato Grosso do Sul, and three from Pará were grouped in the clade of *E. canis*, and four sequences from Pará and all from Rondônia were grouped in the clade of *E. minasensis*. The topologies of phylogenetic trees obtained by both BI (Fig. 7a) and ML (Fig. 7b) methods corroborated the findings. Additionally, ML analysis inferred a subdivision within the *E. canis* clade. Five sequences found in Xenarthra mammals formed a minor clade close to a sequence detected in a dog from Colombia. Four sequences formed a second clade with the other sequences of *E. canis* analyzed. Finally, a sequence obtained from a *M. tridactyla* (83) from São Paulo was positioned separately from the others, with 98% branch support.

**Figure 8:**
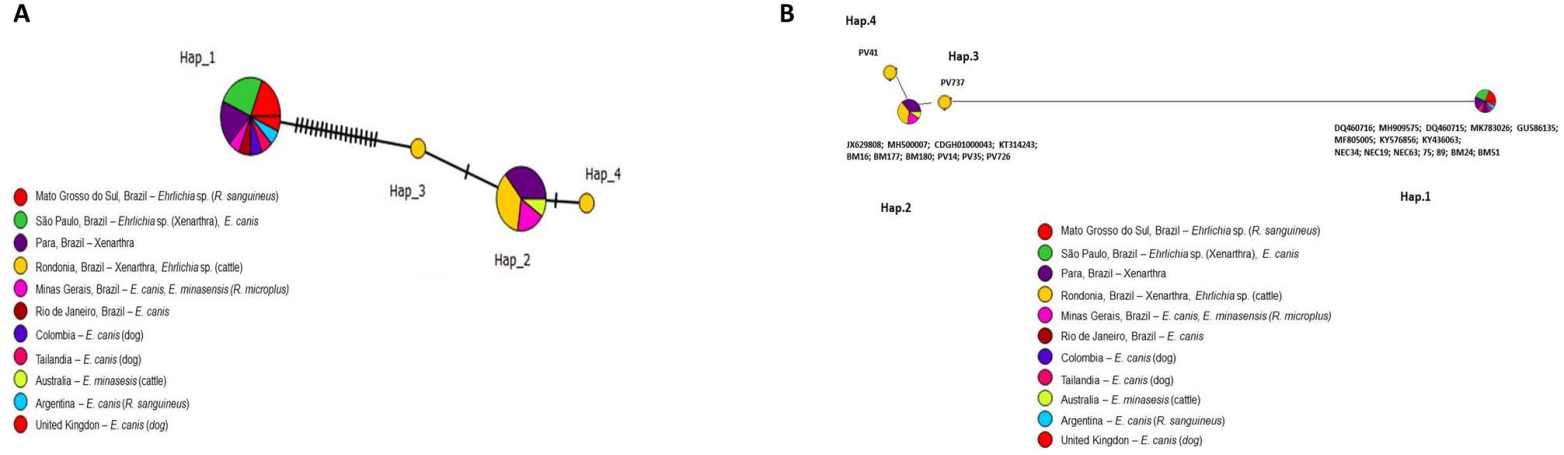
(**A**) TCS network of *dsb* sequences of *E. canis*, *E. minasensis* and those obtained in the present study performed with PopART v.1.7 software (https://popart.otago.ac.nz/index.shtml  (**B**) Split-network performed with Splitstree v4.14.6 software using the parameters “Neighbor-Net and” Uncorrected p-distance (https://en.freedownloadmanager.org/Windows-PC/SplitsTree4-FREE.html.).

Genotypes based on 29 *Ehrlichia* sequences were analyzed. These included 17 sequences obtained in this study as well as *E. canis* and *E. minasensis* sequences detected in different countries. A total of four genotypes were found, with π = 0.03570, hd = 0.569, and K = 9.28079 (Table 5). Genotype #1 comprised all sequences of *E. canis* retrieved from GenBank as well as the sequences detected in Xenarthra sampled in this study in São Paulo and Mato Grosso do Sul, and three sequences from Pará (BM24, BM51, and BM61). Genotype #2 comprised all *E. minasensis* sequences retrieved from GenBank, three Xenarthra sequences sampled in Rondônia, and four from Pará (BM16, BM177, BM180, PV14). Genotypes #3 and #4, on the other hand, comprised unique sequences detected in specimens of *B. variegatus* from Rondônia, PV337, and PV41, respectively. Based on genotype network analysis (Fig. 8a), genotype #1 (*E. canis*) originated from genotype #3 through several mutational events. The latter seems to have originated from genotype #2 (*E. minasensis*), which, in turn, originated from genotype #4, both from a mutational event. The split-network analysis corroborated the main findings described by the genotype network analysis (Fig. 8b).

### Co-positivity for *Anaplasma* spp. and *Ehrlichia* spp.

Of the 147 positive animals, 25 (17%) were co-positive for *Anaplasma* spp. and *Ehrlichia* spp., among 21 sloths (18 *B. variegatus* [12 from Pará and six from Rondônia], one *C. didactylus* from Pará, one *Bradypus* spp. and one *Choloepus* spp. from Rondônia), and four anteaters (three *T. tetradactyla* and one *M. tridactyla* from São Paulo).

## Discussion

The present study revealed a high rate of positivity for Anaplasma spp. and Ehrlichia spp. in Xenarthra mammals sampled in four different Brazilian states. Of the 330 animals, 147 (44.54%) were positive for at least one of the agents. Of these, 25 (17%) were positive for both *Ehrlichia* spp. and *Anaplasma* spp. Until this study, molecular data concerning Anaplasmataceae agents in biological samples of the Superorder Xenarthra have been scant. Guillemi et al.^28^ detected the presence of *A. marginale* morulae in blood smears from a giant anteater in Argentina, which was confirmed by PCR assays based on *msp-5* and *msp1-α* genes. In Brazil, Soares et al.^20^ detected a new *dsb* genotype of *Ehrlichia* sp. in a three-toed sloth (*Bradypus tridactylus*) obtained in the state of Pará. The sequence was allocated a separate clade that was sister to the clade of *E. ruminantium,* and close to the sequences of *Ehrlichia* spp. detected in a horse and a fox in Brazil. The same animal was positive for the *rrs* gene, whose sequence was allocated in a clade close to *A. phagocytophilum*.

The present study reports the occurrence of two possible species of *Anaplasma* spp. in mammals of the Superorder Xenarthra from Brazil, since the two genes analyzed showed low identity values obtained by BLASTn and the phylogenetic findings positioned the sequences obtained in this study in single clades that were separate from the others.

BLAST analyses performed for *Anaplasma* spp. showed that all *rrs* sequences detected in sloths from the states of Rondônia and Pará showed identity values lower < 99% (not exceeding 98.5%) with sequences of *A. ovis*, *A. marginale,* and *A. centrale*. Additionally, the sequences detected in anteaters from São Paulo also showed an identity < 99% with sequences of *A. phagocytophilum* and *A. odocoilei*. However, these same sequences showed an identity > 99% with an *Anaplasma* sequence previously detected in a coati from MS (KY4999186). Previous studies have defined *rrs* sequences as having at least 95% identity to be identified at the genus level and 99% to be identified at the species level^58–60^. In view of this, we have proposed two new species circulating in these animals, and the species detected in São Paulo’s anteaters is probably the same as that found in the Mato Grosso do Sul coatis. The analyses performed in the 23S–5S region intergenic sequences corroborated with the *rrs* gene. The identities obtained were quite low, not exceeding 90%, strengthening the hypothesis of two new species.

The phylogenetic analyses corroborated the BLASTn results. The analysis of the *rrs* sequences of *Anaplasma* spp. obtained from sloths of Rondônia and Pará were allocated close to two *Anaplasma* sequences previously detected in rodents (*R. rattus* and *H. megacephalus*) from Brazil. In addition, the clades formed by these two sequences and those found in the present study had a sister clade formed by *A. marginale*, *A. ovis,* and *A. capra*. The *Anaplasma* sequences detected in anteaters from São Paulo were allocated in a clade close to a new genotype of *Anaplasma* spp. previously detected in wild mammals from the Pantanal Sul-matogrossense.

A similar topology was observed in the phylogeny based on the 23S–5S intergenic region, in which the clade formed by the *Anaplasma* sequences from Xenarthra sampled in Rondônia and Pará was a sister clade that was formed by *Anaplasma* species detected in ruminants. The two *Anaplasma* sequences obtained from anteaters in the state of São Paulo were in a clade completely separated from the other sequences, although they were closer to the *A. phagocytophilum* clade, raising questions about the possible influence of the geographical location or host species on the occurrence of *Anaplasma* species that affect these animals.

Out of the seven samples positive in the qPCR for the *msp2* gene of *A. phagocytophilum*, only one (14.2%) was positive in the PCR based on the 23S–5S region. Despite this, it was phylogenetically related to the clade formed by *A. marginale* and *A. ovis*. Similarly, four samples that were also positive for the *msp2* gene were positioned close to the clade of *A. marginale*, *A. ovis,* and *A. capra* in the phylogenetic analysis based on the *rrs* gene. This potentially indicates the possibility of the aforementioned qPCR protocol to amplify *msp-2* gene fragments from *Anaplasma* species phylogenetically related to *A. phagocytophilum*. Alternatively, the animals may have been co-infected with *A. phagocytophilum* and the new Candidatus species. MSP2, an external membrane protein present in all *Anaplasma* species, is encoded by several polymorphic genes in *A. marginale*, *A. centrale,* and *A. ovis,* and by only one gene in *A. phagocytophilum*^61^.

To better understand the results, genetic diversity analysis and distance genealogies were performed. The results of both corroborated the previously presented phylogenetic positioning. For both the *rrs* gene and the 23S–5S intergenic region, the genotype analyses showed that the *Anaplasma* sequences obtained in Rondônia and Pará states formed new genotypes (#4 and #1, for *rrs* and intergenic regions, respectively), whereas the sequences obtained from anteaters in the SP state comprised one genotype (#8 for *rrs* and intergenic region).

In addition, the genotype network based on the *rrs* gene suggests that the genotype circulating in sloths in Rondônia and Pará states might have originated through three mutational events from genotype #1, which was detected in a *R. rattus* from Brazil, explaining the proximity of both in phylogenetic analysis. The genotype circulating in São Paulo anteaters is the same genotype previously found in a coati in Mato Grosso do Sul, which might have originated through a mutational event from a median vector. Additionally, the genotype network based on the intergenic region suggests that both genotypes (#1 and #8) found in this study might have originated from different median vectors through numerous mutational events. The distance analysis for both genes showed that *Anaplasma* sequences obtained from Xenarthra sampled in the northern region of the country were positioned apart from the others, but were closer to *A. marginale* and *A. ovis*. On the other hand, the sequences obtained from Xenarthra sampled in São Paulo were positioned apart, albeit closer to *A. odocoilei* and *A. phagocytophilum*, based on the *rrs* gene and intergenic region, respectively.

All analyses corroborated and provided strong evidence of the circulation of two new species of *Anaplasma* in Xenarhtra in Brazil, related to the region inhabited by these animals. We propose naming the species circulating in Rondônia and Pará states as ‘*Candidatus* Anaplasma amazonensis’ and the species detected in São Paulo as ‘*Candidatus* Anaplasma brasiliensis’. Further studies are needed to validate these species as well as to determine the vectors responsible for their transmission.

Regarding the findings for *Ehrlichia* spp., all analyses including BLASTn, corroborated and grouped the sequences as *E. canis* or *E. minasensis*. In addition, two sequences detected in sloths phylogenetically close to *E. minasensis* formed two new and distinct genotypes (#3 and #4).

The *rrs genotypes* phylogenetically related to *E. canis* have been increasingly described in wild animals from Brazil, and have already been detected in wild canids^12,14^, wild felids^15^, rodents^14,18,19^, coatis^14^, and geese (*Neochen jubata*)^24^. *E. minasensis*, a recently described species that is genetically similar to *E. canis*^62,63^, can infect and cause clinical signs in cattle in central-western Brazil^64^. It has already been detected in cattle in North America^65^, Ethiopia^66^, Brazil^64^, in cervids in Canada^67^, dogs in Israel^68^, and in several species of ticks^68–74^.

Interestingly, the *dsb* sequences of *E. canis* obtained from Xenarthra mammals comprised animals sampled in the states of São Paulo, Mato Grosso do Sul, and portions of Pará. The *dsb* sequences of *E. minasensis* were obtained from animals from Rondônia and Pará states. Similar to the analysis of *rrs* and 23S–5S *Anaplasma* sequences, these findings again raise questions about the possible regionalization of the *Ehrlichia* and *Anaplasma* species found in Xenarthra from Brazil.

Tick vectors of *E. canis* include *R. sanguineus* sensu lato (tropical lineage) and *D. variabilis*^75–77^. Although DNA from *E. minasensis* has already been detected in tick species that include *Rhipicephalus* (*R. microplus* and *R. sanguineus*)^68,69,72,73^, *Hyalomma* spp.^71,72^, *Haemaphysalis hystricis*^74^, and *Amblyomma sculptum*^72^, the vectorial competence and capability have not been assessed thus far.

The clear dichotomy between the *Ehrlichia* and *Anaplasma* species that infect Xenarthra from different regions of the country may be related to the distribution and abundance of tick species that function as Anaplasmataceae vectors^78^. However, previous studies performed in different Brazilian states have shown that the different tick species that parasitize Xenarthra mammals are more correlated to the host species than to the geographic region. For instance, sloths are mainly parasitized by *Amblyomma varium* and *A. geayi*, with the latter found more in the state of Pará. While giant anteaters and southern anteaters are usually parasitized by A*. nodosum*, *A. sculptum,* and *A. calcaratum*, armadillos are frequently parasitized by *A. pseudoconcolor*, *A. auricularium,* and *A. sculptum*^27, 79–82^. These studies show the high parasitic specificity of some species of ticks that infest these animals. For instance, the ticks collected from anteaters from the state of São Paulo in the present study were all identified as *A. nodosum*, which was the most frequently found species in this group of animals (data not shown).

The vectorial capacity of the tick species frequently found in Xenarthra mammals for Anaplasmataceae agents is still unknown. Although the majority of ticks that parasitize this group of mammals belong to the genus *Amblyomma* spp., the main vectors of *Ehrlichia* spp. in Brazil belong to the genus *Rhipicephalus* spp. Further studies should be conducted to assess the vectorial capacity of these ticks and to better understand the genetic diversity of Anaplasmataceae agents that infect mammals of the Xenarhra superorder in Latin America as well as the possible role of these animals in the epidemiological cycle of these agents.

## Conclusion

The present study showed the high occurrence of *Anaplasma* spp. (27.57%) and *Ehrlichia* spp. (24.54%) in free-living Xenarthra mammals sampled from four states in Brazil. In addition, the study provides the first description of the occurrence of *E. canis* and *E. minasensis* in this group of mammals. The analysis of two genetic regions of *Anaplasma* spp., one conserved (*rrs*), and another one more diverse (intergenic region 23S–5S) revealed similar results of the low identity in BLASTn analysis, phylogenetic positioning in two different clades that were separate from the others containing known species, and formation of two different genotypes from those comprising known *Anaplasma* species by the diversity analysis. Based on these findings, we propose two new *Candidatus* species—‘*Candidatus* Anaplasma amazonensis’ and ‘*Candidatus* Anaplasma brasiliensis’—in Xenarthra from Brazil.

## Supplementary information

Supplementary Table 1.

## Data Availability

The data tha support the findings of this study are openly available in National Center for Biotechnology Information at https://www.ncbi.nlm.nih.gov/, reference number *rrs:* MT199810–MT199833; *dsb*: MT212405–MT212423; 23S–4S intergenic region: MT267341–MT267354.
