# Supplementary material for: Ehrlichia spp. and Anaplasma spp. in Xenarthra mammals from Brazil, with evidence of novel ‘Candidatus Anaplasma spp.’
Source: Sci Rep. 2020 Jul 28;10:12615. doi: 10.1038/s41598-020-69263-w (PMC7387473; doi:10.1038/s41598-020-69263-w)
Supplement: Supplementary file 1 — Supplementary Table 1. [file 41598_2020_69263_MOESM1_ESM.docx]

**Supplementary Material**

**Table 3- Supplementary Material**. Results of efficiency, R^2^, slope and Y-intercept of qPCR assays for Ehrlichia spp. and Anaplasma spp.

| **Gene (Agent)** | **Efficiency Variation (mean)** | **R^2^ Variation (mean)** | **Slope Variation (mean)** | **Y-intercept Variation (mean)** |
| --- | --- | --- | --- | --- |
| *groEL* (*Ehrlichia* spp.*)* | 84.7 - 97% (90.70%) | 0.971 - 0.991 (0.9835) | -3.753 - -3.397 (-3.5715) | 38.762 - 42.30 (40.04) |
| *groEL* (*Anaplasma* spp.) | 100.3 -104.3% (102.6%) | 0.993 - 0.991 (0.991) | -3.314 - -3.224 (-3.261) | 37.461 - 38.61 (38.15) |
| *msp-2* (*A. phagocytophilum)* | 100.1 - 103.5% (101.02%) | 0.991 - 0.998 (0.995) | -3.319 - -3.24 (-3.297) | 38.118 - 39.195 (38.549) |
| *msp1-β* (*A. marginale)* | 97.2 - 104.1% (100.65%) | 0.994 - 0.988 (0.991) | -3.392 - -3.228 (-3.31) | 44.775 - 45.229 (45.002) |
| *dsb (E. canis)* | 99.2 -103.8% (101.5%) | 0.987 - 0.993 (0.99) | -3.341 - -3.235 (-3.288) | 41.645 - 42.256 (41.9505) |
| *vlpt* (*E. chaffeensis)* | 101.5 - 104.6% (103.05%) | 0.908 - 0.975 (0.942) | -3.286 - -3.217 (-3.251) | 42.209 - 43.204 (42.706) |
